# Supplementary material for: Maternal Vaginal Ureaplasma spp. Colonization in Early Pregnancy Is Associated with Adverse Short- and Long-Term Outcome of Very Preterm Infants
Source: Children (Basel). 2021 Apr 3;8(4):276. doi: 10.3390/children8040276 (PMC8066242; doi:10.3390/children8040276)
Supplement: Supplementary file 1 [file children-08-00276-s001.pdf]

Table 1. Short-term clinical outcome of study patients with follow up data at 24 months corrected age according to vaginal *Ureaplasma* spp. colonization.

	**Whole Group** *n* **= 92**	***Ureaplasma* spp. Negative** *n* **= 55**	***Ureaplasma* spp. Positive** *n* **= 37**	***p*-Value**
EOS, *n* (%)	1(1.1)	1 (1.8)	0 (0.0)	0.99
Any IVH, *n* (%)	19 (20.7)	7 (12.7)	12 (32.4)	0.03
Severe IVH, n (%)	6 (6.5)	1	5	0.04
PVL, *n* (%)	2 (2.2)	1 (1.8)	1 (2.7)	0.99
NEC, *n* (%)	7 (7.6)	3	4	0.43
Any ROP, *n* (%)	26 (28.3)	9	17	<0.005
Severe ROP, *n* (%)	6 (6.5)	2	4	0.22
BPD 28 days #, *n* (%)	37 (40.2)	16	21	0.01
BPD 36 weeks #, *n* (%)	12 (13.1)	8	4	0.76

only patients surviving 28 days of life and 36 weeks of adjusted age, respectively, are included.
